# A one-dimensional Hg^II^ coordination polymer based on bis­(pyridin-3-ylmeth­yl)sulfane

**DOI:** 10.1107/S205698901701619X

**Published:** 2017-11-17

**Authors:** Suk-Hee Moon, Youngjin Kang, Ki-Min Park

**Affiliations:** a Department of Food and Nutrition, Kyungnam College of Information and Technology, Busan 47011, Republic of Korea; b Division of Science Education, Kangwon National University, Chuncheon 24341, Republic of Korea; c Research institute of Natural Science, Gyeongsang National University, Jinju 52828, Republic of Korea

**Keywords:** crystal structure, Hg^II^ compound, dipyridyl ligand, zigzag coordination polymer, hydrogen bonding, C—H⋯π inter­actions

## Abstract

The reaction of mercury(II) chloride with bis­(pyridin-3-ylmeth­yl)sulfane (*L*, C_12_H_12_N_2_S) in methanol afforded the title crystalline coordination polymer *catena*-poly[[di­chlorido­mercury(II)]-μ-bis­(pyridin-3-ylmeth­yl)sulfane-κ^2^ *N*:*N*′], [HgCl_2_ *L*]_*n*_. The asymmetric unit consists of one Hg^II^ cation, one *L* ligand and two chloride anions. Each Hg^II^ ion is coordinated by two pyridine N atoms from separate *L* ligands and two chloride anions. The metal adopts a highly distorted tetra­hedral geometry, with bond angles about the central atom in the range 97.69 (12)–153.86 (7)°. Each *L* ligand bridges two Hg^II^ ions, forming an infinite –(Hg–*L*)_*n*_– zigzag chain along the *b* axis, with an Hg⋯Hg separation of 10.3997 (8) Å. In the crystal, adjacent chains are connected by inter­molecular C—H⋯Cl hydrogen bonds, together with Hg—Cl⋯π inter­actions [chloride-to-centroid distance = 3.902 (3) Å], that form between a chloride anion and the one of the pyridine rings of *L*, generating a two-dimensional layer extending parallel to (101). These layers are further linked by inter­molecular C—H⋯π hydrogen bonds, forming a three-dimensional supra­molecular network.

## Abstract 2

The reaction of Hg^II^ with the bridging ligand bis­(pyridin-3-ylmeth­yl)sulfane afforded a one-dimensional zigzag chain polymeric structure, with the charge balanced by two coordinated chloride anions. C—H⋯Cl hydrogen bonds and Hg—Cl⋯π inter­actions, together with C—H⋯π hydrogen bonds, stabilize the crystal structure.

## Chemical context   

The structural topology of coordination polymers generated from the self-assembly of transition metal ions and organic mol­ecules functioning as spacer ligands depends mainly on the structures of the spacer ligands and the coordination geom­etries adopted by the metal ions. The flexibility, length and coordinating ability of the spacer ligands exert strong influences on the formation of coordination polymers and their resulting diverse topologies (Zheng *et al.*, 2009 ▸; Leong & Vittal, 2011 ▸; Liu *et al.* 2011 ▸). For this reason, both rigid and flexible dipyridyl-type spacer ligands with strong coordinating ability and functional characteristics have been widely used to construct a variety of coordination polymers with inter­esting structures and attractive potential applications in material science (Silva *et al.*, 2015 ▸; Furukawa *et al.*, 2014 ▸; Wang *et al.*, 2012 ▸).
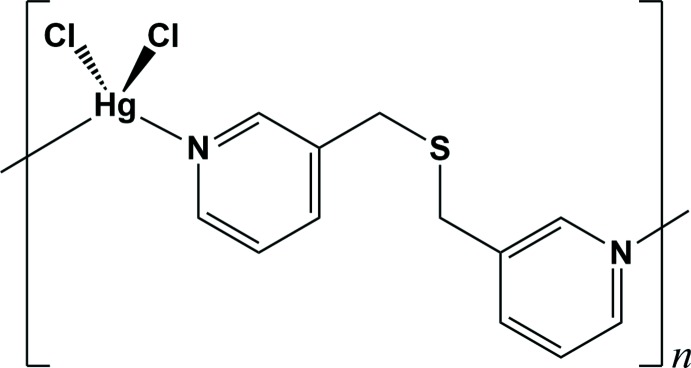


Our group has also synthesized the flexible dipyridyl-type ligand bis­(pyridine-3-ylmeth­yl)sulfane (*L*), and has reported its Ag^I^ and Co^II^ coordination polymers (Moon *et al.*, 2017*a* ▸,*b* ▸). Our continuing inter­est in the development of coordination polymers based on this ligand led us to investigate a coordin­ation polymer with an Hg^II^ cation. The reaction of mercury(II) chloride with *L* (synthesized according to a previously reported procedure: Park *et al.*, 2010 ▸; Lee *et al.*, 2012 ▸) afforded the title compound. Herein, we describe its structure, which involves a one-dimensional zigzag-chain.

## Structural commentary   

**Figure 1:**
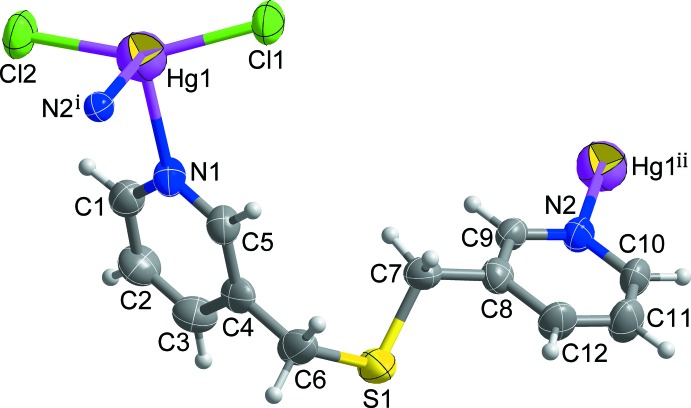
View of the mol­ecular structure of the title compound, showing the atom-numbering scheme [symmetry codes: (i) −*x* + 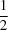, *y* − 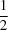, −*z* + 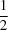; (ii) −*x* + 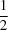, *y* + 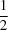, −*z* + 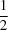]. Displacement ellipsoids are drawn at the 50% probability level.

**Figure 2:**
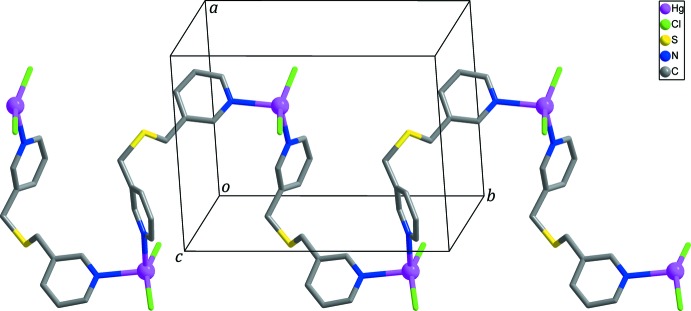
The polymeric zigzag chain propagating along the *b*-axis direction. H atoms are omitted for clarity.

Fig. 1 ▸ shows the mol­ecular structure of the title compound, [Hg*L*Cl_2_]_*n*_, *L* = bis­(pyridine-3-ylmeth­yl)sulfane, C_12_H_12_N_2_S. The asymmetric unit comprises one Hg^II^ cation, one *L* ligand and two chloride anions. The Hg^II^ ion is four-coordinated, binding to two Cl anions and two pyridine N atoms from two separate symmetry-related *L* ligands, forming a highly distorted tetra­hedral geometry (Fig. 1 ▸), with the tetra­hedral angles falling in the range of 97.69 (12)–153.86 (7)° (Table 1 ▸). The S atoms of the *L* ligands are surprisingly not bound to the soft Hg^II^ cations. Each *L* ligand bridges two Hg^II^ cations, resulting in an infinite zigzag chain propagating along the *b-*axis direction (Fig. 2 ▸). The separation between the Hg^II^ ions in the chain is 10.3997 (8) Å. In the *L* ligand, the dihedral angle between the two terminal pyridine rings is 78.52 (18)°, and the flexible thio­ether moiety [C4–C6–S1–C7–C8] shows a bent arrangement with a *gauche-*-*anti* configuration [C4—C6—S1—C7 = 71.9 (5)°; C6—S1—C7—C8 = 172.1 (5)°]. The conformation of the *L* ligand, along with its N_py_—Hg—N_py_ coordination angle [98.39 (16)°], may induce the zigzag topology of the chain.

## Supra­molecular features   

**Figure 3:**
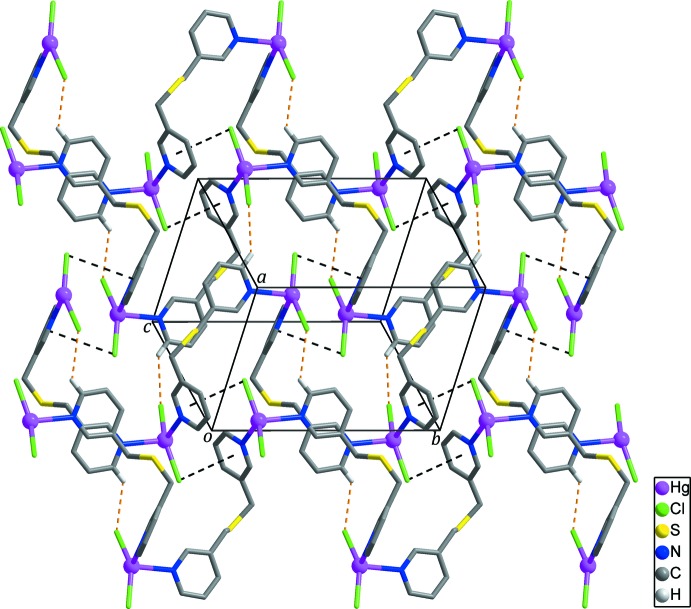
The layer formed through inter­molecular C–H⋯Cl hydrogen bonds (yellow dashed lines) and Hg—Cl⋯π inter­actions (black dashed lines) between the zigzag chains. H atoms not involved in inter­molecular inter­actions are omitted for clarity.

**Figure 4:**
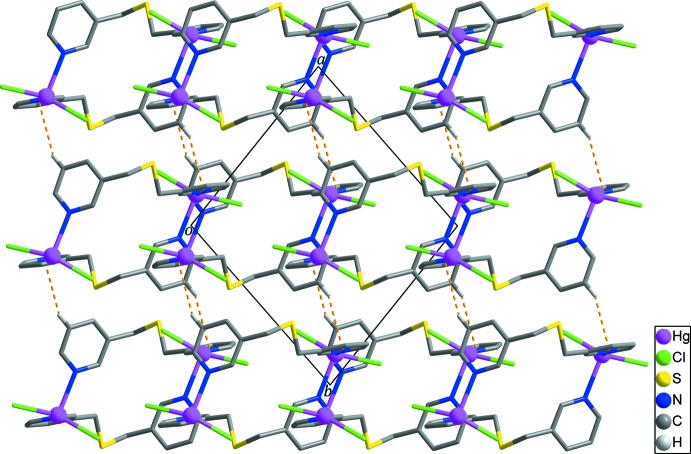
The three-dimensional supra­molecular network generated by inter­molecular C—H⋯π inter­actions (yellow dashed lines) between the layers of polymer chains. H atoms not involved in inter­molecular inter­actions are omitted for clarity.

In the crystal structure, adjacent zigzag chains are connected by C10—H10⋯Cl1 hydrogen bonds (Fig. 3 ▸, Table 2 ▸) and Hg—Cl⋯π inter­actions (Chifotides & Dunbar, 2013 ▸; Matter *et al.*, 2009 ▸) between the chloride anions and the pyridine rings of *L* with Cl2⋯*Cg*1^iv^ = 3.902 (3) Å and Hg1—Cl2⋯*Cg*1^iv^ = 77.21 (6)° [Fig. 3 ▸; *Cg*1 is the centroid of the N1/C1–C5 ring; symmetry code: (iv) −*x* + 1, −*y* + 1, −*z* + 1], generating layers extending parallel to (101). Neighboring layers are linked by C2—H2⋯*Cg*2 hydrogen bonds (Table 2 ▸; Fig. 4 ▸), resulting in the formation of a three-dimensional supra­molecular network.

## Database survey   

A search of the Cambridge Structural Database (Version 5.38, update May 2017; Groom *et al.*, 2016 ▸) for the title ligand (*L*) gave three hits. Two (REJCAL, RENHOI; Hanton *et al.*, 2006 ▸) are copper(I) iodide coordination polymers adopting staircase- and loop-type structures, respectively. The other (EXEZOW; Seo *et al.*, 2003 ▸) is a cyclic dimer-type silver(I) BF_4_ complex. Recently, our group has also reported the crystal structures of silver(I) (Moon *et al.*, 2017*a* ▸) and cobalt(II) (Moon *et al.*, 2017*b* ▸) NO_3_ coordination polymers that display twisted ribbon- and loop-type topologies, respectively. In these complexes, the flexible thio­ether moiety (C_py_–C–S–C–C_py_) of the *L* ligand adopts a bent arrangement that is similar to that of the Hg^II^ polymer described here. However, the title compound displays a zigzag topology and is the first example of an Hg^II^ coordination polymer with the ligand *L*.

## Synthesis and crystallization   

The *L* ligand was synthesized according to a literature method (Park *et al.*, 2010 ▸; Lee *et al.*, 2012 ▸). Crystals of the title compound were obtained by slow evaporation of a methanol solution of *L* with HgCl_2_ in a 1:1 molar ratio.

## Refinement   

Crystal data, data collection and structure refinement details are summarized in Table 3 ▸. All H atoms were positioned geometrically and refined as riding: C—H = 0.93 Å for C*sp* ^2^—H and 0.97 Å for methyl­ene C—H with *U*_iso_(H) = 1.2*U*_eq_(C).

## Supplementary Material

Crystal structure: contains datablock(s) I, New_Global_Publ_Block. DOI: 10.1107/S205698901701619X/sj5541sup1.cif

Structure factors: contains datablock(s) I. DOI: 10.1107/S205698901701619X/sj5541Isup2.hkl

CCDC reference: 1584773

Additional supporting information:  crystallographic information; 3D view; checkCIF report

## Figures and Tables

**Table 1:** Selected geometric parameters (Å, °)

Hg1—Cl1	2.3610 (16)	Hg1—N2^i^	2.434 (5)
Hg1—Cl2	2.3751 (16)	Hg1—N1	2.436 (5)
			
Cl1—Hg1—Cl2	153.86 (7)	Cl1—Hg1—N1	97.69 (12)
Cl1—Hg1—N2^i^	100.29 (12)	Cl2—Hg1—N1	97.91 (13)
Cl2—Hg1—N2^i^	98.03 (12)	N2^i^—Hg1—N1	98.39 (16)

Symmetry code: (i) 
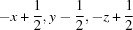
.

**Table 2:** Hydrogen-bond geometry (Å, °) *Cg*2 is the centroid of the N2/C8–C12 ring.

*D*—H⋯*A*	*D*—H	H⋯*A*	*D*⋯*A*	*D*—H⋯*A*
C10—H10⋯Cl1^ii^	0.93	2.80	3.526 (6)	136
C2—H2⋯*Cg*2^iii^	0.93	2.89	3.689 (7)	145

Symmetry codes: (ii) 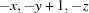; (iii) 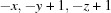.

**Table 3:** Experimental details

Crystal data	
Chemical formula	[HgCl_2_(C_12_H_12_N_2_S)]
*M*_r_	487.79
Crystal system, space group	Monoclinic, *P*2_1_/*n*
Temperature (K)	298
*a*, *b*, *c* (Å)	10.4724 (11), 13.1128 (14), 10.8914 (12)
β (°)	100.1171 (18)
*V* (Å^3^)	1472.4 (3)
*Z*	4
Radiation type	Mo *K*α
μ (mm^−1^)	10.94
Crystal size (mm)	0.45 × 0.40 × 0.30

Data collection	
Diffractometer	Bruker SMART APEX CCD
Absorption correction	Multi-scan (*SADABS*; Bruker, 2014 ▸)
*T*_min_, *T*_max_	0.447, 0.746
No. of measured, independent and observed [*I* > 2σ(*I*)] reflections	8706, 3197, 2413
*R*_int_	0.047
(sin θ/λ)_max_ (Å^−1^)	0.639

Refinement	
*R*[*F* ^2^ > 2σ(*F* ^2^)], *wR*(*F* ^2^), *S*	0.033, 0.076, 1.03
No. of reflections	3197
No. of parameters	163
H-atom treatment	H-atom parameters constrained
Δρ_max_, Δρ_min_ (e Å^−3^)	0.62, −1.62

Computer programs: *APEX2* and *SAINT* (Bruker, 2014 ▸), *SHELXS97* and *SHELXTL* (Sheldrick, 2008 ▸), *SHELXL2014* (Sheldrick, 2015 ▸), *DIAMOND* (Brandenburg, 2010 ▸) and *publCIF* (Westrip, 2010 ▸).

## supplementary crystallographic information

### Crystal data

**Table:**

[HgCl_2_(C_12_H_12_N_2_S)]	*F*(000) = 912
*M**_r_* = 487.79	*D*_x_ = 2.200 Mg m^−^^3^
Monoclinic, *P*2_1_/*n*	Mo *K*α radiation, λ = 0.71073 Å
*a* = 10.4724 (11) Å	Cell parameters from 9216 reflections
*b* = 13.1128 (14) Å	θ = 2.5–28.2°
*c* = 10.8914 (12) Å	µ = 10.94 mm^−^^1^
β = 100.1171 (18)°	*T* = 298 K
*V* = 1472.4 (3) Å^3^	Plate, colorless
*Z* = 4	0.45 × 0.40 × 0.30 mm

### Data collection

**Table:**

Bruker SMART APEX CCD diffractometer	2413 reflections with *I* > 2σ(*I*)
φ and ω scans	*R*_int_ = 0.047
Absorption correction: multi-scan (SADABS; Bruker, 2014)	θ_max_ = 27.0°, θ_min_ = 2.5°
*T*_min_ = 0.447, *T*_max_ = 0.746	*h* = −13→11
8706 measured reflections	*k* = −16→10
3197 independent reflections	*l* = −13→13

### Refinement

**Table:**

Refinement on *F*^2^	0 restraints
Least-squares matrix: full	Hydrogen site location: inferred from neighbouring sites
*R*[*F*^2^ > 2σ(*F*^2^)] = 0.033	H-atom parameters constrained
*wR*(*F*^2^) = 0.076	*w* = 1/[σ^2^(*F*_o_^2^) + (0.0315*P*)^2^] where *P* = (*F*_o_^2^ + 2*F*_c_^2^)/3
*S* = 1.03	(Δ/σ)_max_ < 0.001
3197 reflections	Δρ_max_ = 0.62 e Å^−^^3^
163 parameters	Δρ_min_ = −1.62 e Å^−^^3^

### Special details

**Table:**

Geometry. All esds (except the esd in the dihedral angle between two l.s. planes) are estimated using the full covariance matrix. The cell esds are taken into account individually in the estimation of esds in distances, angles and torsion angles; correlations between esds in cell parameters are only used when they are defined by crystal symmetry. An approximate (isotropic) treatment of cell esds is used for estimating esds involving l.s. planes.

### Fractional atomic coordinates and isotropic or equivalent isotropic displacement parameters (Å^2^)

**Table:**

	*x*	*y*	*z*	*U*_iso_*/*U*_eq_	
Hg1	0.62237 (2)	0.33807 (2)	0.42736 (2)	0.04266 (10)	
Cl1	0.54910 (16)	0.36205 (14)	0.21164 (15)	0.0559 (4)	
Cl2	0.75516 (17)	0.37587 (15)	0.62207 (16)	0.0633 (5)	
S1	−0.06074 (16)	0.35443 (14)	0.33023 (18)	0.0598 (5)	
N1	0.4127 (5)	0.3509 (4)	0.4938 (4)	0.0416 (12)	
N2	−0.1449 (4)	0.6533 (4)	0.0651 (4)	0.0408 (12)	
C1	0.4063 (6)	0.3940 (5)	0.6036 (6)	0.0467 (15)	
H1	0.4823	0.4178	0.6525	0.056*	
C2	0.2918 (7)	0.4044 (5)	0.6469 (6)	0.0551 (17)	
H2	0.2897	0.4384	0.7215	0.066*	
C3	0.1800 (7)	0.3642 (5)	0.5789 (7)	0.0537 (17)	
H3	0.1018	0.3697	0.6079	0.064*	
C4	0.1849 (6)	0.3153 (4)	0.4666 (6)	0.0414 (14)	
C5	0.3037 (6)	0.3121 (4)	0.4282 (6)	0.0425 (14)	
H5	0.3081	0.2812	0.3523	0.051*	
C6	0.0678 (6)	0.2659 (5)	0.3898 (7)	0.0545 (17)	
H6A	0.0341	0.2151	0.4405	0.065*	
H6B	0.0944	0.2307	0.3201	0.065*	
C7	0.0153 (6)	0.4169 (5)	0.2132 (6)	0.0462 (15)	
H7A	0.0896	0.4559	0.2536	0.055*	
H7B	0.0457	0.3660	0.1605	0.055*	
C8	−0.0790 (5)	0.4860 (5)	0.1354 (5)	0.0403 (13)	
C9	−0.0627 (5)	0.5903 (4)	0.1356 (5)	0.0379 (13)	
H9	0.0090	0.6181	0.1873	0.045*	
C10	−0.2482 (6)	0.6139 (5)	−0.0097 (6)	0.0464 (15)	
H10	−0.3048	0.6575	−0.0601	0.056*	
C11	−0.2726 (6)	0.5132 (5)	−0.0141 (6)	0.0574 (17)	
H11	−0.3457	0.4882	−0.0663	0.069*	
C12	−0.1897 (6)	0.4473 (5)	0.0583 (6)	0.0503 (16)	
H12	−0.2068	0.3776	0.0563	0.060*	

### Atomic displacement parameters (Å^2^)

**Table:**

	*U*^11^	*U*^22^	*U*^33^	*U*^12^	*U*^13^	*U*^23^
Hg1	0.03985 (13)	0.04678 (16)	0.03812 (15)	0.00356 (11)	−0.00208 (9)	−0.00140 (12)
Cl1	0.0515 (9)	0.0733 (12)	0.0385 (9)	0.0022 (8)	−0.0039 (7)	−0.0006 (8)
Cl2	0.0602 (10)	0.0726 (12)	0.0482 (10)	−0.0080 (9)	−0.0154 (8)	−0.0059 (9)
S1	0.0373 (8)	0.0776 (13)	0.0666 (12)	0.0107 (8)	0.0149 (8)	0.0249 (10)
N1	0.041 (3)	0.048 (3)	0.035 (3)	0.011 (2)	0.006 (2)	−0.002 (2)
N2	0.039 (3)	0.042 (3)	0.039 (3)	−0.004 (2)	0.000 (2)	0.002 (2)
C1	0.047 (3)	0.046 (4)	0.046 (4)	−0.003 (3)	0.004 (3)	0.001 (3)
C2	0.063 (4)	0.061 (5)	0.045 (4)	0.004 (3)	0.021 (3)	−0.009 (3)
C3	0.056 (4)	0.053 (4)	0.057 (4)	−0.001 (3)	0.024 (3)	−0.002 (3)
C4	0.043 (3)	0.033 (3)	0.049 (4)	0.011 (2)	0.009 (3)	0.012 (3)
C5	0.044 (3)	0.047 (4)	0.037 (3)	0.010 (3)	0.007 (3)	−0.003 (3)
C6	0.044 (4)	0.047 (4)	0.071 (5)	−0.001 (3)	0.008 (3)	0.017 (3)
C7	0.039 (3)	0.051 (4)	0.051 (4)	0.000 (3)	0.013 (3)	0.006 (3)
C8	0.043 (3)	0.047 (4)	0.031 (3)	0.001 (3)	0.006 (2)	−0.003 (3)
C9	0.032 (3)	0.045 (4)	0.036 (3)	−0.005 (2)	0.003 (2)	−0.002 (3)
C10	0.040 (3)	0.055 (4)	0.039 (4)	−0.005 (3)	−0.006 (3)	0.001 (3)
C11	0.056 (4)	0.055 (4)	0.053 (4)	−0.013 (3)	−0.011 (3)	−0.004 (3)
C12	0.051 (4)	0.040 (4)	0.056 (4)	−0.014 (3)	−0.002 (3)	−0.003 (3)

### Geometric parameters (Å, º)

**Table:**

Hg1—Cl1	2.3610 (16)	C4—C5	1.381 (8)
Hg1—Cl2	2.3751 (16)	C4—C6	1.504 (9)
Hg1—N2^i^	2.434 (5)	C5—H5	0.9300
Hg1—N1	2.436 (5)	C6—H6A	0.9700
S1—C6	1.810 (6)	C6—H6B	0.9700
S1—C7	1.813 (6)	C7—C8	1.490 (8)
N1—C5	1.335 (8)	C7—H7A	0.9700
N1—C1	1.336 (7)	C7—H7B	0.9700
N2—C9	1.334 (7)	C8—C9	1.378 (8)
N2—C10	1.339 (7)	C8—C12	1.402 (8)
N2—Hg1^ii^	2.434 (5)	C9—H9	0.9300
C1—C2	1.370 (8)	C10—C11	1.345 (9)
C1—H1	0.9300	C10—H10	0.9300
C2—C3	1.376 (10)	C11—C12	1.372 (9)
C2—H2	0.9300	C11—H11	0.9300
C3—C4	1.390 (9)	C12—H12	0.9300
C3—H3	0.9300		
			
Cl1—Hg1—Cl2	153.86 (7)	C4—C6—S1	113.9 (4)
Cl1—Hg1—N2^i^	100.29 (12)	C4—C6—H6A	108.8
Cl2—Hg1—N2^i^	98.03 (12)	S1—C6—H6A	108.8
Cl1—Hg1—N1	97.69 (12)	C4—C6—H6B	108.8
Cl2—Hg1—N1	97.91 (13)	S1—C6—H6B	108.8
N2^i^—Hg1—N1	98.39 (16)	H6A—C6—H6B	107.7
C6—S1—C7	98.7 (3)	C8—C7—S1	110.2 (4)
C5—N1—C1	117.9 (5)	C8—C7—H7A	109.6
C5—N1—Hg1	123.0 (4)	S1—C7—H7A	109.6
C1—N1—Hg1	119.0 (4)	C8—C7—H7B	109.6
C9—N2—C10	118.8 (5)	S1—C7—H7B	109.6
C9—N2—Hg1^ii^	123.3 (4)	H7A—C7—H7B	108.1
C10—N2—Hg1^ii^	117.9 (4)	C9—C8—C12	116.7 (6)
N1—C1—C2	122.3 (6)	C9—C8—C7	122.2 (5)
N1—C1—H1	118.8	C12—C8—C7	121.0 (6)
C2—C1—H1	118.8	N2—C9—C8	123.1 (5)
C1—C2—C3	119.3 (6)	N2—C9—H9	118.4
C1—C2—H2	120.3	C8—C9—H9	118.4
C3—C2—H2	120.3	N2—C10—C11	121.9 (6)
C2—C3—C4	119.4 (6)	N2—C10—H10	119.0
C2—C3—H3	120.3	C11—C10—H10	119.0
C4—C3—H3	120.3	C10—C11—C12	120.1 (6)
C5—C4—C3	117.0 (6)	C10—C11—H11	120.0
C5—C4—C6	120.6 (6)	C12—C11—H11	120.0
C3—C4—C6	122.4 (6)	C11—C12—C8	119.3 (6)
N1—C5—C4	123.9 (6)	C11—C12—H12	120.4
N1—C5—H5	118.1	C8—C12—H12	120.4
C4—C5—H5	118.1		
			
C5—N1—C1—C2	−3.7 (9)	C6—S1—C7—C8	172.1 (5)
Hg1—N1—C1—C2	179.6 (5)	S1—C7—C8—C9	114.8 (5)
N1—C1—C2—C3	3.8 (10)	S1—C7—C8—C12	−65.0 (7)
C1—C2—C3—C4	−1.1 (10)	C10—N2—C9—C8	−0.1 (8)
C2—C3—C4—C5	−1.4 (9)	Hg1^ii^—N2—C9—C8	176.9 (4)
C2—C3—C4—C6	177.5 (6)	C12—C8—C9—N2	−1.4 (9)
C1—N1—C5—C4	0.9 (9)	C7—C8—C9—N2	178.9 (5)
Hg1—N1—C5—C4	177.5 (4)	C9—N2—C10—C11	1.2 (9)
C3—C4—C5—N1	1.6 (9)	Hg1^ii^—N2—C10—C11	−175.9 (5)
C6—C4—C5—N1	−177.4 (5)	N2—C10—C11—C12	−0.7 (10)
C5—C4—C6—S1	−117.0 (5)	C10—C11—C12—C8	−0.8 (10)
C3—C4—C6—S1	64.1 (7)	C9—C8—C12—C11	1.8 (9)
C7—S1—C6—C4	71.9 (5)	C7—C8—C12—C11	−178.5 (6)

Symmetry codes: (i) −*x*+1/2, *y*−1/2, −*z*+1/2; (ii) −*x*+1/2, *y*+1/2, −*z*+1/2.

### Hydrogen-bond geometry (Å, º)

*Cg*2 is the centroid of the N2/C8–C12 ring.

**Table:**

*D*—H···*A*	*D*—H	H···*A*	*D*···*A*	*D*—H···*A*
C10—H10···Cl1^iii^	0.93	2.80	3.526 (6)	136
C2—H2···*Cg*2^iv^	0.93	2.89	3.689 (7)	145

Symmetry codes: (iii) −*x*, −*y*+1, −*z*; (iv) −*x*, −*y*+1, −*z*+1.
